# Metal–Organic Frameworks (MOFs) and Their Derivatives for Environmental Remediation and Energy Devices

**DOI:** 10.3390/ma19122531

**Published:** 2026-06-11

**Authors:** Raghavendra P. Bakale, Sushant S. Kakati, Shridhar N. Mathad, Leena V. Hublikar, Amita Somya, Anish Khan, Khalid A. Alzahrani, Malik Abdul Rub, Naved Azum

**Affiliations:** 1 Department of Chemistry, Jain College of Engineering, Belagavi 590001, Karnataka, India; 2 Department of Engineering Physics, East Point College of Engineering and Technology, Bangalore 560049, Karnataka, India; 3 Department of Physics, School of Advanced Sciences, KLE Technological University, Hubballi 580020, Karnataka, India; 4 Department of Chemistry, School of Advanced Sciences, KLE Technological University, Hubballi 580020, Karnataka, India; 5 Department of Chemistry, Amity School of Applied Sciences, Amity University, Bangalore 562110, Karnataka, India; somya.amita@gmail.com; 6 Center of Excellence for Advanced Materials Research, King Abdulaziz University, Jeddah 21589, Saudi Arabia; 7 Chemistry Department, Faculty of Science, King Abdulaziz University, Jeddah 21589, Saudi Arabia

**Keywords:** metal–organic frameworks, environmental remediation, energy devices, adsorption, catalysis, electrocatalysis

## Abstract

Metal–organic frameworks (MOFs) are crystalline porous materials made of metal nodes coordinated by organic linkers. Their high surface areas, tunable pore sizes, adjustable chemical environments, and modular design make MOFs promising for two main application domains: environmental remediation and energy conversion or storage. In this review, we explore the applications of both newly designed MOFs and MOF-derived materials. These applications include catalysis, electrocatalysis, sensing, pollutant removal, batteries, supercapacitors, and other hybrid energy devices. We attempt to correlate MOF structure with key parameters, such as metal centers, ligands, defects, and porosity, to performance. We also discuss the future use of MOFs in real-world devices. This depends on overcoming challenges such as scalability, conductivity, stability, and environmental safety.

## 1. Introduction

**Figure 1:**
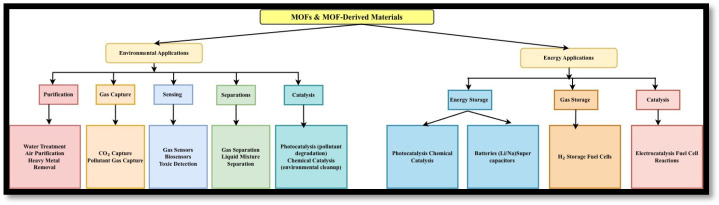
Broad environmental and energy applications of MOFs and MOF-derived materials, including purification, gas capture, sensing, separations, catalysis, and energy storage.

Over the past two decades, metal–organic frameworks (MOFs) have garnered immense research interest due to their unique combination of large internal surface area, tunable porosity, and chemical versatility [1,2,3,4,5]. Originally explored for gas storage and separation, MOFs are increasingly targeted toward environmental remediation by leveraging their photochemical properties, including photocatalytic activity and photoluminescence (removing or degrading pollutants, sensing, membrane separations) and energy applications, as their high surface area enables the effective adaptation of considerable amounts of charge carriers, augmenting their potential for capacitive charge storage devices and various categories of batteries (electrochemical energy storage, electrocatalysis, photochemical/electrochemical devices) [6,7,8,9,10]. As global environmental challenges increase water pollution, greenhouse gas emissions, energy demands, etc., the functional materials such as MOFs offer opportunities for more efficient, selective, and modular solutions [11,12,13,14]. This review is intended to provide equal emphasis on environmental and energy aspects, as depicted in Figure 1. We begin by summarizing synthetic/design strategies and structure–property relationships, then proceed to environmental remediation applications, energy device applications, and finally, MOF-derived materials. We also discuss practical concerns, challenges, and conclude with perspectives and recommendations for advancing the field.

**Figure 2:**
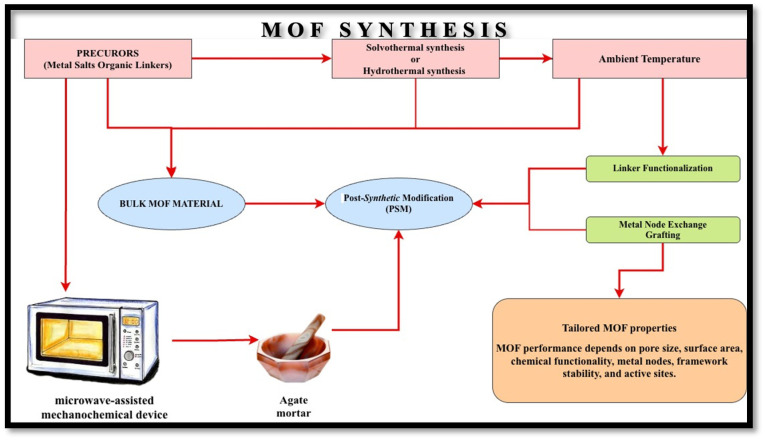
Various synthetic pathways of MOFs and post-synthetic modifications.

Solvothermal [15] or hydrothermal synthesis [16] remains the standard approach for making highly crystalline MOFs. This method frequently uses modulators to control crystal growth, defect densities, and particle size. When harsh conditions might degrade sensitive functional groups, or scalability is a concern, ambient or room-temperature assembly is preferred [17]. Alternative methods, such as microwave-assisted, mechanochemical, and electrochemical syntheses (Figure 2), are gaining attention. These methods enable faster, more energy-efficient, and environmentally friendly processing [18]. Besides substituting metal nodes and functionalizing organic linkers, external catalytic and sensing moieties can be grafted onto MOFs [19,20]. Such post-synthetic modifications play a crucial role. Typical synthetic routes are shown in Figure 2.

Various principles govern the structural tuning of MOFs. The exceptional water stability of UiO-66, a Zr-based MOF, is due to the selection of the proper metal node, which affects the redox properties, coordination environment, and hydrolytic stability [21]. It is found that the fine-tuning of chemical environments, hydrophilicity, and interactions with catalytic intermediates or pollutants is because of the linker design, which includes variations in linker rigidity, length, and functional groups such as –NO_2_, –OH, –NH_2_, or heterocycles, that determine the overall functionality and the structure [22]. Other strategies, such as introducing mixed-metal/mixed-linker systems, enhance performance by enabling electronic structure adjustments and the creation of multifunctional frameworks [23]. In catalytic and adsorption processes, improved accessibility, enhanced reaction kinetics, and increased active-site density are achieved through defect engineering and the introduction of porosity via controlled missing-cluster or missing-linker defects [24,25].

Metal–organic frameworks, through chemical modification and structural transformation, have led to numerous MOF derivatives. MOF-derived carbons (MDCs) have been obtained by the pyrolysis or carbonization processes [26]. Materials with enhanced electrical conductivity and superior mechanical or electrochemical stability are formed by embedding metal and metal oxide nanoparticles within conductive carbon matrices [27]. Metal oxides, phosphides, sulfides and other related compounds that preserve the original MOF morphology are formed by the controlled treatments such as oxidation, phosphidation, sulfidation, and reduction [28]. For applications in battery electrodes that require improvements in mass diffusion and volume accommodation, hollow or yolk–shell architectures can be achieved by selectively removing framework components [29]. Precisely controlled coordination environments for MOF structures are developed by single-atom catalysts (SACs), which stabilize isolated metal atoms within carbon matrices derived from MOFs [30].

The structural features of MOFs, such as the metal nodes, organic linkers, pore size, coordination geometry, presence of various functional groups, strength of metal–linker coordination, framework flexibility, crystallite size and defect concentration, topology, and incorporation of dopants, affect MOF properties such as surface area, stability, adsorption capacity, catalytic activity, and mechanical robustness. The pore size distribution, with <2 nm micropores accounting for 92% of the total surface area, provides a high surface area essential for strong adsorption, and 2–50 nm mesopores enhance mass transport [31]. Properties such as selectivity, adsorption, gas interactions, and catalytic activity are also strongly influenced by the presence of open metal sites, polar functional groups, and acid–base centers [32]. The properties such as redox behavior, thermal stability and hydrolytic nature of MOFs, which are required for energy storage and catalytic applications, are governed by the strength of metal–linker coordination [33]. The enhancement in the charge transport and conductivity of MOFs is typically achieved by incorporating conductive guests/dopants, conductive-supported hybridization, conjugated linkers, or converting MOFs into their conductive derivatives [34]. The performance and stability of MOFs are enhanced by smaller crystallites, which improve diffusion dynamics, and the defect concentration is strategically controlled so that defects can create new active or binding sites, without compromising structural integrity [35]. Table 1 establishes the fundamental relationship between the structural features of MOFs and their resulting physicochemical properties. Translating these intrinsic characteristics into practical solutions requires a targeted approach. The knowledge of how the elements like pore size, defect concentration, and metal–linker coordination dictate stability and catalytic activity provides the necessary foundation for designing materials that are suited to the specific real-world challenges. Building on this structural framework, Table 2 bridges the gap between fundamental properties and practical utility. It summarizes how the specific MOFs that are characterized by the unique stability profiles, surface areas, and linker compositions can be strategically selected as well as deployed for the targeted environmental and industrial applications, ranging from catalytic processes and gas capture to pollutant removal and water purification.

### Effect of Changing Linker from BDC to Amino BDC

**Figure 3:**
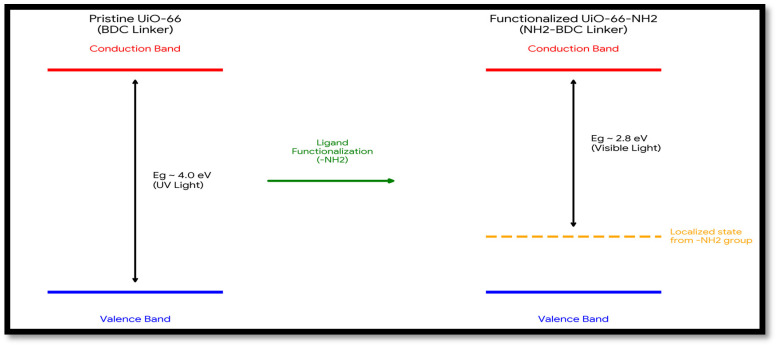
Electronic Structure Scheme: UiO-66 vs. UiO-66-NH_2_.

To illustrate the profound impact of ligand engineering on electronic properties, the transition from pristine UiO-66 to its amine-functionalized derivative, UiO-66-NH_2_, serves as a primary example. In pristine UiO-66, constructed with standard terephthalate (BDC) linkers, the highest occupied molecular orbital (HOMO) is primarily dominated by the O 2p and C 2p orbitals of the organic ligand, while the lowest unoccupied molecular orbital (LUMO) is localized on the empty Zr 4d orbitals of the metal-oxo cluster. This configuration results in a wide intrinsic band gap of approximately 4.0 eV, restricting its light absorption to the ultraviolet (UV) region (wavelengths < 310 nm) and severely limiting its solar energy utility. However, substituting the BDC linker with 2-aminoterephthalate (NH_2_-BDC) fundamentally alters this electronic structure. The strongly electron-donating primary amine (–NH_2_) group introduces N 2p non-bonding electrons into the aromatic system. This functionalization creates a new, localized highest occupied state that sits energetically higher than the original valence band of the pristine framework, while the conduction band minimum (localized on the Zr clusters) remains relatively unchanged. Consequently, this upward shift of the valence band edge quantitatively reduces the optical band gap from ~4.0 eV to roughly 2.8 eV (as shown in Figure 3).

This specific narrowing shifts the material’s optical absorption edge from the UV spectrum into the visible light region (~440 nm). By successfully bridging this energy gap, the functionalized UiO-66-NH_2_ framework can generate significantly more electron-hole pairs under standard solar irradiation, directly resulting in the vastly improved visible-light photocatalytic performance frequently reported for environmental remediation and solar fuel generation.

## 2. Methodology

We conducted a comprehensive literature review of scientific publications on metal–organic frameworks (MOFs) and their derivatives for environmental remediation and energy applications. Our search included original research articles and review papers from databases such as Scopus and Web of Science. We used keywords like Metal-organic frameworks, environmental remediation, adsorption, catalysis, energy devices, and electrocatalysis. Some figures in this study were modified using draw.io, which we used only to organize, optimize, and present experimental workflows. All scientific content, experimental design, data interpretation, and intellectual contributions were developed and verified by the authors. We did not use any AI-generated scientific data, analytical results, or textual conclusions in preparing the manuscript. The use of draw.io (V30.1.2) was limited to improving the clarity, formatting, and quality of the figures.

## 3. Environmental Applications of MOFs

**Figure 4:**
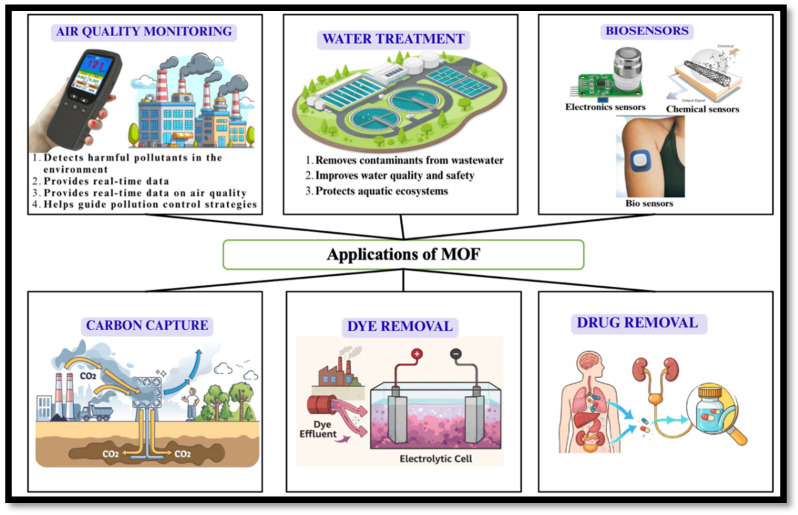
Environmental Applications of MOFs.

MOFs possess properties that help restore the environment, which suffers from various forms of pollution, including air, soil, water, and food pollution [49,50,51,52]. They are used to detect heavy metals, toxic gases, contaminants and many more using highly sensitive sensors and remove harmful pollutants by means of selective separation, adsorption and degradation of hazardous chemicals through catalytic reactions by virtue of their properties like tunable pore structures, high porosity, and functionalized surfaces [53,54]. Table 3 gives a glimpse of different applications of metal–organic frameworks (MOFs), along with the properties that influence their performance, their distinguishing features, and representative examples of MOFs often employed, with corresponding literature references. It helps link MOF characteristics with uses, enabling identification of their suitability across varied areas such as sensing, water treatment, gas storage, catalysis, and biomedical applications (Figure 4).

### 3.1. Adsorption and Separation

**Figure 5:**
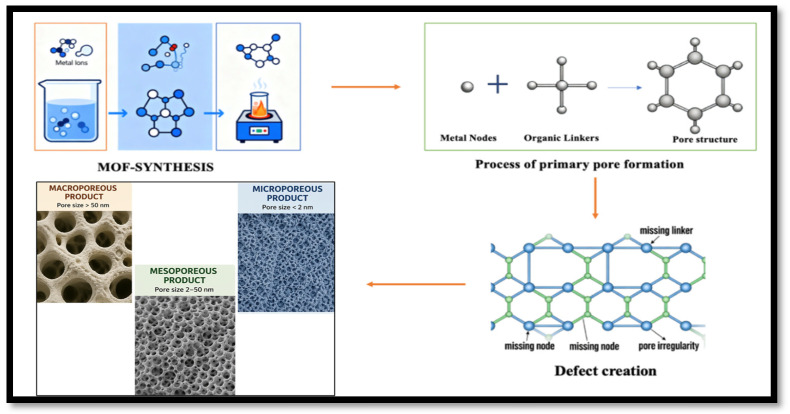
Sketches show the MOF synthesis, process of primary pore formation, pore scales and defect creation.

Adsorption- and separation-based processes using MOFs play an important role in environmental remediation [67]. The properties of MOFs (Figure 5) and chemically functionalized frameworks significantly enhance their adsorption performance in the removal of heavy metals and organic pollutants [68]. This could be due to electrostatic attraction, hydrogen bonding, coordination bonding, and π-π interactions between the amino and hydroxyl functional groups on MOFs and contaminants, including cadmium, lead, arsenic, and chromium (Table 4). The combined effect of enhanced selectivity with high adsorption capacity and structural stability of MOF-modified membranes and MOF composites is found to improve removal efficiencies for pharmaceutical residues in water and dyes as well. Amine functionalized and open metal sites containing MOFs have demonstrated high CO_2_/N_2_ selectivity and CO_2_ capture capacity in gas capture and separation [69], while for the capture of NO_x_, SO_x_ and volatile organic compounds (VOCs), from industrial emissions, MOFs are being developed through pore size tuning and amine grafting technique [70]. For the applications that use membranes to enable gas purification while reducing fouling and selective separations for desalination, thin-film MOF membranes or mixed-matrix membranes (MMMs) formed by combining MOFs and polymers have been utilized [71], as the surface functionalized MOFs are found to enhance the compatibility with polymer matrices, which in turn minimizes interfacial defects and improves membrane performance [72]. MOFs have potential for practical environmental remediation applications such as catalysis, pollutant adsorption, and sensing, despite challenges such as stability, cost, and scalability.

### 3.2. Catalytic Degradation of Pollutants

Metal–organic frameworks (MOFs) based on metals such as Ti or Fe, or composites with semiconductors, are most suitable for photocatalytic degradation of organic pollutants due to their ability to absorb UV or visible light [78]. Reactive oxygen species (ROS), such as superoxide and hydroxyl radicals, formed by charge separation, light-induced electron excitation, and migration to surface reaction sites during the photocatalytic process in MOFs can degrade pollutants, such as dyes and antibiotics [79]. Ti-based MOFs like MIL-125, Zr-based MOF like UiO-66, and Zn-based MOF-5 behave like semiconductors and form heterojunctions within composite materials, which enhance charge separation, thereby improving the photocatalytic efficiency of MOFs [80]. MOF-derived materials containing Fe activate H_2_O_2_ and degrade stubborn organics (Fenton-like catalysis), with some of the derivatives that embed iron oxide nanoparticles in carbon matrices, imparting better stability and reusability [81]. MOFs can undergo oxidation, producing reactive oxygen species that are effective for water treatment applications [82]. Some MOFs and their derivatives can catalyze pollutant transformations, such as nitrate reduction or organic contaminant oxidation, through photoelectrochemical and electrocatalytic processes [83,84,85]. Currently, the focus of ongoing research is on charge-carrier separation, light-absorption enhancement, and stability, which can make MOF-based photocatalysts workable for environmental remediation.

### 3.3. Sensing and Monitoring

Metal–organic frameworks (MOFs), due to their potent luminescent properties and tunable cavities, can be used to detect trace amounts of nitroaromatics, heavy metals, and gases [86]. The emission of luminescent MOFs (LMOFs) containing guest dyes or fluorescent linkers can be modulated by analyte binding through mechanisms such as cation exchange, energy absorption, or framework interactions [87]. These LMOFs exhibit high sensitivity and selectivity toward certain metal ions, such as Hg^2+^, Pb^2+^, Fe^3+^, Cu^2+^, and Cr(VI) anions, with detection limits reaching ppb levels [88]. MOF-polymer composites with their improved compatibility and reduced interfacial defects with polymer matrices are used in Chemi resistive or electrochemical sensors, which offer increased sensor recovery times and cycling stability [89]. Novel Cd^2^-based LMOFs exhibit excellent performance in detecting Fe^3+^ cations and Cr(VI) anions with low detection limits and high selectivity [90]. Thus, MOF-based conductive sensors and luminescent materials are found to be selective, rapid, and reusable for environmental monitoring of heavy metals and other hazardous substances.

### 3.4. Antimicrobial and Disinfection Applications

Metal–organic frameworks (MOFs), because of their ability to incorporate and release metal ions known for their antimicrobial properties, such as silver (Ag), copper (Cu), and zinc (Zn), exhibit antimicrobial and disinfection applications [91]. MOFs can act through direct interactions between positively charged metal ions and negatively charged bacterial membranes, leading to membrane disruption and, eventually, bacterial death [92]. Even MOFs may catalyze the generation of reactive oxygen species (ROS), which further damage microbial cells [93]. MOFs come into good contact with microbes present in both water and air, making them effective in filters and coatings designed to inhibit bacteria and viruses [94]. However, antimicrobial efficacy is maintained while minimizing potential toxicity by controlling the release or leaching of metal ions [95]. Recently, MOFs have been used as carriers, synergistically with other materials, for the sustained release of antimicrobial agents to improve activity and stability [96]. Overall, MOFs are found to be most suitable for antimicrobial applications due to their ROS generation, metal ion release, physical entrapment, and pathogen filtration.

## 4. Energy Device Applications of MOFs

MOFs are used in numerous energy device applications, including energy storage using batteries combined with supercapacitors, in efficient energy conversion with scalable storage using electrolysis and fuel systems and in photoelectrochemical cells for solar-driven chemical reactions [97,98,99]. Apart from that, in Table 5, a comparison of other energy device applications is provided.

### 4.1. Batteries

**Figure 6:**
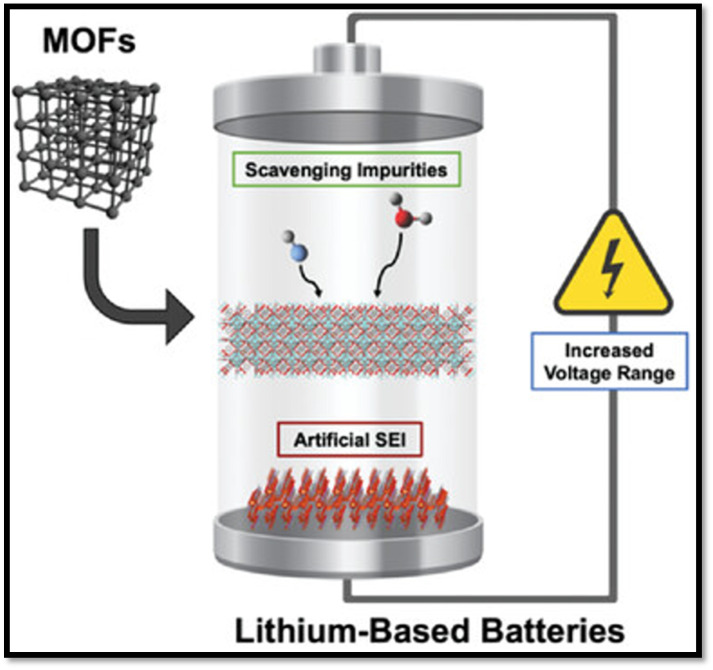
Schematic representations of MOF-derived porous carbon electrodes illustrating enhanced porosity, conductivity, and stability, and their integration with carbonized MOFs as single-atom catalysts for high-performance lithium–sulfur (Li–S) batteries [115].

As one of the most important components in energy storage devices, many metal–organic frameworks (MOFs) and their derivatives have been found to be potent electrode materials [106,107,108], especially in batteries (Figure 6). Most of the MOFs that are converted into metal oxides, phosphides or sulfides and even carbonized MOFs effectively serve as anode or cathode materials [109]. The hierarchical pore structures of MOFs allow improved ionic diffusion, volume changes to be accommodated during charge–discharge cycles, and increased interface area between the electrode and electrolyte, thereby enhancing battery performance [110]. In the case of lithium–sulfur (Li–S) batteries, it is very important to reduce the shuttle effect, which hampers cycle life and compromises safety. MOF-derived porous carbons are more effective at confining sulfur and trapping polysulfides [111]. Further improvements in cycling stability and safety can be achieved by homogenizing lithium deposition through advanced designs, such as ultrathin MOF nanosheets and separators modified with single-atom arrays derived from the MOFs [112]. Even MOF-derived single-atom catalysts, such as Co–N–P on carbon, are used to enhance reaction kinetics and improve the chemical adsorption of active species, such as iodine and polysulfides, in aqueous batteries [113]. To optimize electrode performance in the battery, MOFs must be tuned for crystallinity, porosity, and chemical properties. However, challenges remain in long-term structural stability and in improving conductivity, which are required for their commercial applications [114].

### 4.2. Supercapacitors

**Figure 7:**
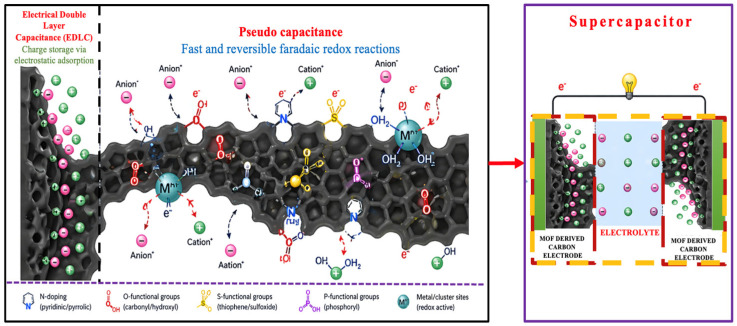
Schematic illustrating the role of MOF-derived carbons in introducing pseudo capacitance in supercapacitors.

Metal–organic framework (MOF)-derived carbon materials are highly promising for supercapacitor electrodes due to their high surface area, tunable pore structures, and the possibility of heteroatom doping (e.g., nitrogen, sulfur, phosphorus), which enhances conductivity and electrochemical performance [116], as shown in Figure 7. MOFs, through their microporous and mesoporous surface, facilitate electric double-layer capacitance, which promotes effective transport and ion adsorption [117]. MOF-derived carbons embedded with metal or metal oxide nanoparticles impart pseudo-capacitance, significantly boosting capacitance and energy density and not being restricted to pure physical storage [118]. Recent studies of asymmetric supercapacitors that are constructed with MO-derived carbons and metal oxides from materials such as MIL-100 (Fe) demonstrate delivery of high specific capacitances (127.4 F/g at 0.1 A/g), excellent power density, superior energy density (25.5 Wh/kg), and cycling stability with retention of 90% capacity over thousands of cycles [119]. Furthermore, remarkable cyclic stability and capacitance retention at very high current densities are achieved by nitrogen-doped carbon nanostructures derived from the flexible MOF [120]. Though there are challenges related to the intrinsic low conductivity of MOFs, they can be overcome by forming a composite, doping with heteroatoms, and incorporating metal oxides, which help outperform conventional supercapacitor materials [121].

### 4.3. Electrocatalysis

**Figure 8:**
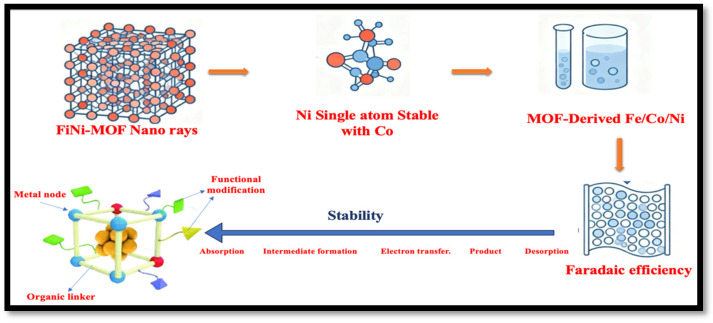
Representation of FiNi-MOF nanoarrays evolving into Ni single-atom stabilized MOF-derived Fe/Co/Ni catalysts.

**Figure 9:**
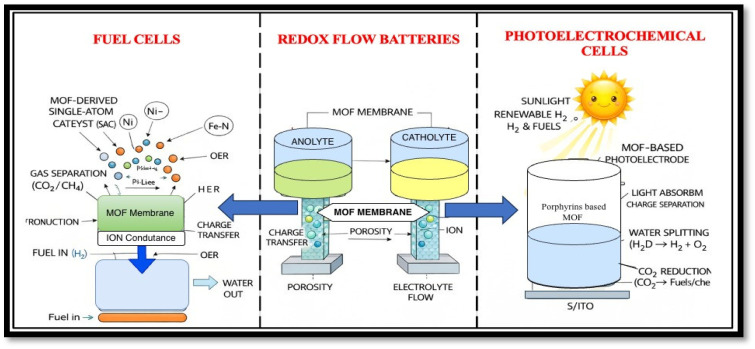
MOF-based materials in fuel cells, redox flow batteries, and photoelectrochemical cells comparison.

Metal–organic frameworks (MOFs) and their derivatives like conductive carbon matrices embedded with metal/metal oxide nanoparticles or single-atom catalysts show excellent performance in electrocatalysis despite limitations in intrinsic conductivity [122] (Figure 8). Some of the recent advances include outstanding oxygen evolution reaction (OER) catalytic activity of the bimetallic FeNi-MOF nanoarrays, which require low overpotentials at high current densities (around 239 mV at 50 mA/cm^2^) and exhibit remarkable long-term stability over hundreds of hours [123]. Here, the transitions in iron’s oxidation state accelerate reaction kinetics, enhancing catalytic activity [124]. In the case of MOF-derived Ni single-atom catalysts, a Faradaic efficiency of around 67% for carbon monoxide production from electrolytic bicarbonate conversion at 100 mA/cm^2^ is achieved with mesoporous structures (pore size around 35 nm), surpassing many silver nanoparticle catalysts [125]. MOF-derived Fe, Co, and Ni catalysts, when optimized through defect engineering, morphology control, and doping strategies, demonstrated low overpotentials for both the hydrogen evolution reaction (HER) and the oxygen evolution reaction (OER), which are useful for water splitting [126]. Apart from that, MOF-based materials are also useful in fuel cells as electrocatalysts for ORR and as membranes for selective gas permeability or ion conduction [127]. MOFs and their derivatives have appeared as suitable catalysts and structural components in fuel cell research [128]. For efficient fuel cell operations, electrochemical reactions play a very critical role. MOF-derived single-atom catalysts (SACs) have shown remarkable selectivity, catalytic activity, and stability, which lead to efficient ORR, HER, and OER [129]. MOF-derived atomically dispersed metal sites within carbon matrices are durable, cost-effective, and scalable in next-generation fuel cells [130]. Not only limited to catalysis, but MOFs are also developed as membrane materials for selective gas permeability, ion conduction, and superior selectivity, which address defect issues and grain boundaries present in crystalline MOF membranes [131]. The tunable pore sizes and structural integrity of MOF under operational conditions make them appropriate materials for ion transport and efficient gas separation in both industrial gas purification systems and fuel cell applications [132]. In photoelectrochemical (PEC) cells for solar-driven fuel production, MOFs are integrated into light-harvesting electrodes [133] (Figure 9). In order to drive CO_2_ reduction under illumination and water splitting, transition-metal MOFs immobilized on silicon substrates (based on porphyrin) are utilized for efficient charge separation and visible-light absorption [134]. Recent MOF architectures, such as Al_2_(OH)_2_CoTCPP@Si, have achieved reduced recombination losses and improved solar-to-fuel conversion efficiencies by influencing p-type silicon for photoexcitation, charge transfer, and catalysis [135]. Further research into hybrid photocatalyst designs is required solely because of their limitations in long-term stability and overall efficiency [136].

## 5. MOF-Derived Materials

### 5.1. MOF-Derived Carbons (MDCs)

**Figure 10:**
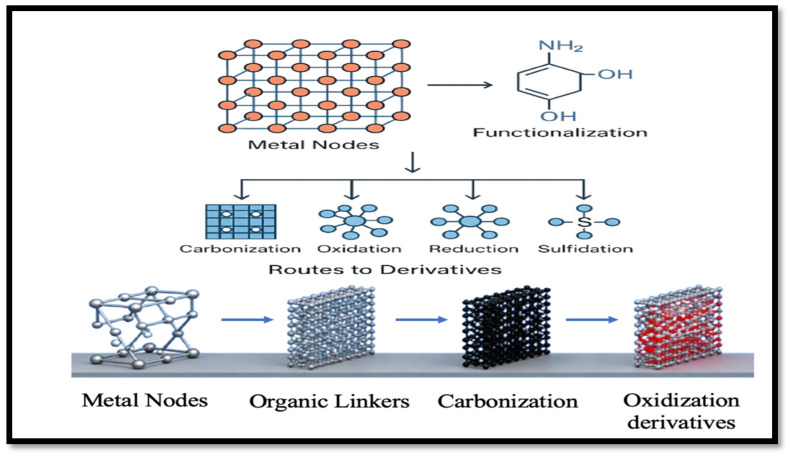
Schematic of MOF scaffold: metal nodes + organic linkers + functionalization; routes to derivatives (carbonization, oxidation, etc.).

Carbonization of MOFs under an inert atmosphere at high temperatures leads to porous carbon materials termed metal–organic framework-derived carbons (MDCs) [137]. By the carbonization process, parent MOF’s structure can be preserved to varying degrees, which produces highly porous frameworks with pore size distribution and controllable morphology [138] (Figure 10). These types of materials with large pore volumes and exceptionally high specific surface areas (up to 10,000 m^2^/g) enable high adsorption capacity and efficient molecular transport [139]. MDCs possess enhanced electrical conductivity, improved catalytic activity, and increased wettability due to their inherent heteroatom doping (nitrogen, sulfur, phosphorus, or boron atoms) retained from the MOF’s organic linkers/metal precursors [140]. Apart from that, these carbon structures show excellent stability in harsh chemical environments, such as acidic or basic media, and chemical and mechanical stability [141]. The choice of the original MOF precursor and carbonization parameters, such as diverse morphologies (e.g., hollow spheres, hierarchical networks, or nanorods), is possible in MDCs, offering tunability for specific applications [142]. These MDCs are now widely used as adsorbents for pollutants and CO_2_ due to their high surface reactivity, as well as electrode materials in supercapacitors, lithium/sodium-ion batteries, and lithium–sulfur batteries, where hierarchical porosity promotes ion diffusion and charge storage efficiency [143]. Furthermore, MDCs provide conductive, chemically stable scaffolds for dispersing metallic nanoparticles or anchoring single atoms, thereby forming active catalysts or supports for electrocatalytic reactions such as oxygen reduction and hydrogen evolution reactions [144]. Overall, MOF-derived carbons represent a versatile class of high-performance, tunable materials bridging the properties of both crystalline MOFs and traditional carbon frameworks for energy, catalysis, and environmental technologies [145].

### 5.2. Architectures of MOFs and Their Derivatives

Through pyrolysis and subsequent treatments such as oxidation, sulfidation, or phosphidation, metal–organic frameworks (MOFs) can be transformed into composites comprising metal, metal oxide, sulfide, or phosphide nanoparticles finely embedded in carbon matrices. The morphology of the MOF is preserved while yielding uniformly dispersed nanoparticles with a highly porous carbon framework, and a large active surface area is maintained through high dispersion, thereby enhancing durability and catalytic performance [146]. Carbon matrix enhances electrical conductivity and mechanical integrity, providing structural stability for catalytic and electrochemical processes [147]. Synergistic effects between the conductive carbon and the reactive metal centers, arising from interfaces between metal nanoparticles and the carbon matrix, create highly active catalytic sites. For instance, synergistic effects of metal nanoparticles embedded in nanoporous carbon-derived frameworks exhibit strong magnetic properties, superior water-treatment and electrocatalytic performance [148,149]. Nanoparticles embedded in carbon matrices exhibit low overpotentials and excellent durability for hydrogen evolution reactions, demonstrating their ability to boost reactivity. Using this route, nanocomposites are designed with tunable composition, structure, and catalytic activity for various applications [150].

### 5.3. Single-Atom Catalysts (SACs)

MOF-derived single-atom catalysts are materials that are made by converting metal–organic frameworks into porous supports with isolated metal atoms. Each atom functions as an independent active site, maximizing atom utilization, improving selectivity, and delivering higher turnover frequencies than nanomaterial-based catalysts [151].

Single metal atoms can be immobilized and stabilized by strong metal-N or metal-O coordination bonds through the carbonization of MOFs, which provides a conductive, high-surface-area, and defect-rich carbon framework [152]. Catalytic performance can be optimized for reactions like the HER, ORR, and CO_2_ reduction reaction (CO_2_RR) by adjusting the coordination environments, such as forming asymmetric M–N_x_–C sites or symmetric M–N_4_ sites, which allows fine-tuning of the electronic structure and geometric configuration of the active centers [153]. MOF-derived single-atom catalysts are found to outperform noble-metal systems. For instance, Ni single-atom catalysts with mesoporous structures have demonstrated roughly better Faradaic efficiency, surpassing many nanomaterial-based catalysts [154]. Additionally, SACs derived from MOFs have proven highly effective in fuel cells and metal–air batteries applications [155]. Overall, MOF-derived single-atom catalysts offer tailored active sites, robust durability, and high selectivity for catalysis, particularly in electrocatalytic energy conversion and CO_2_ utilization technologies [156].

### 5.4. Engineered MOF Structures for Efficient Energy Storage and Catalysis

Advanced metal–organic frameworks (MOFs) structural designs, such as hierarchical, hollow, yolk–shell, and composite architectures, show better performance in catalysis and energy storage systems [157]. In battery electrodes, the most commonly encountered mechanical stress and volume changes are mitigated by hollow and yolk–shell architectures, which provide internal void spaces that buffer expansion and contraction during cycling [158]. They also improve conductivity and stability by enhancing reaction kinetics by shortening ion and electron diffusion paths [159]. For example, in lithium–sulfur batteries, yolk–shell-structured Co–N–C@N-doped carbon spheres are reported to offer uniform cobalt active sites, high conductivity, and enhanced sulfur storage [160].

Efficient ion and mass transport across multiple scales in hierarchical porous architectures is enabled by the integration of micropores, mesopores, and macropores within a single framework [161]. Because of this multilevel porosity, active sites are easily accessible, and diffusion is accelerated, leading to enhanced gas separation and catalytic activity. There is significantly improved kinetics in CO_2_ capture and reaction processes due to 50% higher mass transfer coefficients in hierarchical Cu-MOFs compared to non-hierarchical MOFs [162]. Multifunctionality in composite systems, such as MOF–graphene, MOF–polymer, and MOF–MXene hybrids, is achieved by combining their complementary properties; for instance, MXene/MOF composites exhibit exceptional structural stability, electrical conductivity, and ion diffusion efficiency [163]. One of the studies has demonstrated that MOF-derived CoFe_2_O_4_ nanoparticles integrated with Ti_3_C_2_T_x_ MXene and carbon nanofibers formed a multidimensional network and achieved high capacity retention in lithium-ion battery applications due to improved charge transfer kinetics and mechanical robustness [164]. Electrochemical stability is further enhanced by inhibiting oxidation, thereby preventing structural degradation between MXene surfaces and MOF ligands, providing enhanced conductivity, structural adaptability and improved transport performance across multiple length scales [165,166].

## 6. Practical Considerations

### 6.1. Stability

The stability of metal–organic frameworks (MOFs) is a deciding factor in their practical use. Since many MOFs degrade upon exposure to aqueous or humid environments, their hydrolytic stability is a major concern [167]. Commonly preferred MOFs for aqueous or humid applications are those with titanium–oxygen (Ti-O) or zirconium–oxygen (Zr-O) linkages as they demonstrate notably higher stability due to strong metal–oxygen bonds [168]. MOFs can be specifically engineered for thermal and chemical stability, with robust metal nodes and linkers that are resistant to oxidizing agents, extreme acidic or basic pH, and repeated redox cycling, which can lead to framework degradation [169]. Operational stability of MOFs requires maintaining their structural integrity, resisting fouling, and retaining adsorption or catalytic activity over repeated cycles for their practical applications in energy devices like supercapacitors or batteries, or in continuous pollutant removal [170]. A key challenge in designing MOFs and their derivatives to enhance resistance to environmental stresses and mechanical wear and to achieve extended cycling durability can be addressed through composite formation, defect engineering, or surface modifications [171].

### 6.2. Conductivity

The challenge associated with the low electrical conductivity of most traditional MOFs is a significant bottleneck for their direct use in energy applications and it can be strategically addressed through two primary paradigms: intrinsic (through-bond/through-space) and extrinsic (guest-promoted) enhancement strategies.

Intrinsic conductivity relies on the rational molecular design of the framework structure itself to facilitate continuous charge transport. A primary approach is the incorporation of highly conjugated or pi-conjugated organic linkers that enable extensive electron delocalization throughout the framework [171]. Specific examples of this strategy are beautifully illustrated by 2D conductive MOFs based on extended pi-systems, such as triphenylene (e.g., using hexaiminotriphenylene to form M3(HITP)2 or metal-phthalocyanine (Pc) macrocycles). These planar linkers promote strong in-plane metal–ligand orbital overlap and out-of-plane pi–pi stacking, establishing robust pathways for charge mobility. This intrinsic conductivity can be further optimized during synthesis by utilizing mixed-valence metal ions and soft-donor ligands (e.g., substituting oxygen with sulfur or nitrogen) to maximize the covalency of the metal–ligand bonds [171,172].

Conversely, extrinsic (guest-promoted) conductivity strategies bypass the need to alter the fundamental MOF lattice by introducing external charge carriers. This includes the infiltration of redox-active guest molecules (such as TCNQ) or conductive polymers directly into the MOF pores to form interconnected charge transport pathways. Additionally, integrating MOFs with conductive supports like graphene [172], or embedding conductive fillers such as metal or metal oxide nanoparticles [173], creates highly efficient macroscopic electron networks. Alternatively, high-temperature carbonization can transform MOFs into highly conductive carbon derivatives that retain the structural features and highly tunable porosity of the parent template [174,175].

However, the pursuit of enhanced charge transport requires navigating a critical design compromise. There is an inherent trade-off between electrical conductivity and specific surface area. The introduction of dense, heavily conjugated linker systems (like phthalocyanines), or the extensive loading of framework pores with conductive guests, inevitably reduces the accessible void fraction and the overall BET surface area.

Overall, the conductivity limitations of MOFs can be effectively overcome by carefully balancing these intrinsic and extrinsic strategies—combining molecular design at the node and linker level with post-synthetic modifications or composite formation, ultimately tailoring the porosity–conductivity trade-off to suit specific applications in electrocatalysis, supercapacitors, batteries, and sensors [176,177].

### 6.3. Scalability and Manufacturing

Scalability and large-scale manufacturing of metal–organic frameworks (MOFs) is not easy [178]. The difficulty in scaling up batch solvothermal synthesis methods is mainly due to limitations in reactor size and in controlling reproducibility and uniformity [179]. Presently, the methods under consideration to address this are mechanochemical methods, continuous-flow synthesis, and green solvent-based approaches because of their scalability, sustainability, and cost-effectiveness [180]. Apart from this, these methods enable better control over crystal size, defect levels and morphology while reducing energy consumption and solvent use [181].

Normally, high temperatures and energy-intensive processes associated with post-synthetic treatments, such as pyrolysis, doping, and the formation of MOF derivatives, often contribute to higher production costs and environmental impacts [182]. Therefore, it is crucial to optimize the above-mentioned steps to lower energy consumption and improve efficiency for industrial viability. To produce MOFs in forms suitable for applications such as coatings, membranes, and structured catalysts, some recent advances also focus on shaping techniques, including extrusion, 3D printing, and spray drying [183]. Recently, some companies have been able to achieve commercial-scale production of MOFs, tailoring MOF properties to specific customer needs, thereby demonstrating the feasibility of scaling [184].

### 6.4. Environmental and Health Safety

There are MOFs that exhibit high stability and reduced leaching due to strong metal-linked bonds, thus being useful for water purification. However, many MOFs degrade under real-world conditions, such as ionic strength, fluctuating pH, and the presence of competing species, leading to reduced stability and unexpected leaching of toxic metals during water treatment applications and contaminating the water, posing environmental and health safety issues [185,186,187].

While recent MOF derivatives show higher stability, their performance under complex operational conditions differs quietly from controlled lab settings; this discrepancy highlights the importance of realistic testing of MOFs to understand their potential environmental impacts [188]. There are only limited studies on the life-cycle analysis (LCA) of MOFs that address regeneration processes, end-of-life disposal, and environmental impacts associated with large-scale use. Proper life-cycle analysis of MOFs will improve their efficiency in pollutant removal and address environmental safety concerns [189].

## 7. Challenges and Open Research Questions

A key challenge with metal–organic frameworks (MOFs), which limits their use in energy-related applications such as batteries, electrocatalysis, and sensors, is their low electrical conductivity, which is often below 10^−10^ S/cm [190]. The following are some of the strategies to improve conductivity:

- Conjugated or π-conjugated organic linkers can be used to enable electron delocalization and extended conjugation with metal nodes [191], and mixed valence metal ions or soft-donor ligands can be employed to facilitate electron hopping and charge transfer [192].
- Space-charge transport can be enhanced via π-π stacking between adjacent organic linkers [193] and by incorporating electroactive guest molecules into MOF pores [194].
- By hybridizing conductive materials like graphene, conductive polymers, or metal nanoparticles [195] or by converting MOFs into conductive derivatives by carbonization or embedding metal/metal oxide nanoparticles [196].

Several pathways, like metal–ligand bonding, metal and ligand π system, space π-π interactions, and redox hopping, are involved in the charge transport in conductive MOFs [197]. Due to the insulating nature of most organic linkers and framework structures, despite progress, balancing high porosity with electrical conductivity remains difficult [198]. Hence, current research focuses on designing co-ligand systems and novel linkers, incorporating guest molecules, defect engineering, and optimizing composite formation to overcome the above limitations, as these advances are critical for harnessing MOFs’ full potential in electronic and electrochemical devices.

## 8. Future Outlook and Recommendations

Toxicity and Secondary Pollution Limitations: While MOFs exhibit extraordinary pollutant removal capacities, their widespread deployment in environmental remediation faces a critical, often-overlooked hurdle: the risk of secondary pollution via framework degradation. It is fundamentally counterproductive to remediate water or soil utilizing materials that ultimately leach toxic metal nodes or hazardous organic linkers into the ecosystem.

Metal Node Leaching: The ecotoxicity of a MOF is intrinsically linked to its thermodynamic stability and constituent metal. Frameworks constructed from heavy or transition metals such as Cu, Co, Ni, and Pb pose severe environmental risks; their dissolution in aquatic systems releases free metal ions that are highly toxic to aquatic flora and fauna, often causing severe oxidative stress and disrupting local aquatic food webs. Consequently, for environmental applications, the field must strictly pivot toward robust frameworks utilizing bio-benign nodes, such as Fe (e.g., MIL-53), Zr (e.g., UiO-66), Ti, or Ca.

Linker Toxicity and Degradation Products: Equally critical is the fate of the organic linkers upon framework hydrolysis. Many benchmark MOFs rely on linkers that are not environmentally benign. A prominent example is the zeolitic imidazolate framework ZIF-8, which, despite its popularity, suffers from hydrolytic instability under acidic conditions. The degradation of ZIF-8 in water and soil matrices releases its constituent linker, 2-methylimidazole. Recent toxicological studies have identified imidazole derivatives as highly mobile in aqueous environments and potentially toxic to aquatic life, with concerns regarding cytotoxicity and endocrine disruption. Similarly, the widespread use of terephthalic acid (BDC) in UiO and MIL series poses questions regarding long-term accumulation and microbial degradation pathways in soil ecosystems.

Nano-toxicity: Furthermore, the push toward nano-sized MOFs (nMOFs) to maximize surface area introduces classic nanomaterial toxicity paradigms. In aquatic environments, nMOFs (<100 nm) exhibit high mobility and can readily permeate cellular membranes of aquatic microorganisms and filter-feeders (e.g., Daphnia magna). The intracellular degradation of these nanoparticles can trigger massive reactive oxygen species (ROS) bursts, leading to lipid peroxidation and DNA damage.

**Figure 11:**
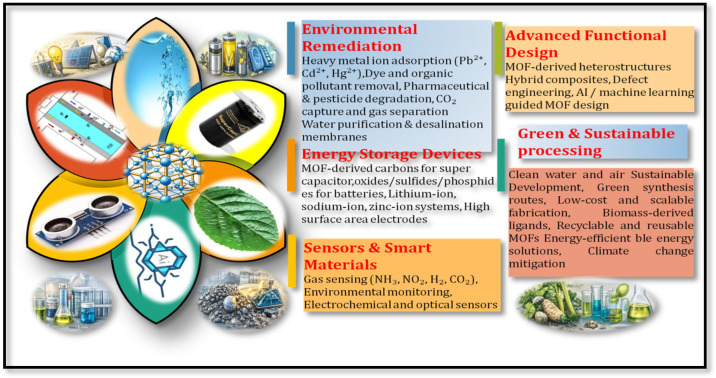
Current Market Trends and Future Scope Of MOF.

Therefore, before any large-scale environmental deployment, it is imperative that newly synthesized MOFs undergo rigorous, standardized ecotoxicity assessments (e.g., OECD guidelines for aquatic and terrestrial toxicity) to definitively map the ecological fate of both their metal nodes and their organic degradation products. Metal–organic frameworks (MOFs) are advancing towards real-world applications by virtue of their multifunctionality [199,200]. Even the current market trends shown in Figure 11 indicate steady growth in gas storage and separation [201], catalysis, environmental remediation [202], healthcare [203], food packaging [204], and the energy sector [205]. The following are the key recommendations to accelerate MOF implementation:

- Design of MOFs for applications emphasizing green, scalable synthesis methods to enhance sustainability and industrial scalability.
- Developing multifunctional systems that integrate adsorption, catalytic degradation, sensing, or combined energy capture to maximize utility and efficiency.
- Improving selectivity, turnover frequency, and reducing precious metal use.
- Comprehensive life-cycle assessments and environmental safety studies addressing metal leaching, degradation product toxicity, disposal, and regeneration for sustainable deployment.

## 9. Conclusions

Metal–organic frameworks (MOFs) and their derivatives are now seen as top choices for important applications such as environmental cleanup, energy conversion, and energy storage. They stand out because they have very high surface areas, tailored pores, adjustable chemical environments, and a modular design that allows easy changes in function. Recent developments have led to the development of MOF-based materials, including single-atom catalysts, finely mixed metal/carbon composites, and other useful structures. These materials show strong potential in areas such as catalysis, electrocatalysis, sensing, pollutant removal, batteries, supercapacitors, and hybrid energy devices. Even with these advances, it is still hard to use MOF-based systems outside the lab. The main problems are high production costs, challenges in scaling up production, low natural electrical conductivity, stability concerns, and questions about environmental safety. To overcome these issues, researchers need to test performance under real conditions, better understand how these materials work, and develop scalable, sustainable methods to make them. Solving these problems is key to moving MOF-based materials from research to real-world use.

## Figures and Tables

**Table 1:** Key structural features of metal–organic frameworks (MOFs) and their influence on functional properties.

Sl. No	Structural Feature	Key Description	Impact on Properties	Reference
1	Micro pores: High surface volume ratio.	Micropores (<2 nm) in this microscopic channel provide a high surface area.	Its high surface volume ratio provides strong adsorption, which aids in gas storage (CO_2_, H_2_), large molecule absorption (VOCs), reduces transport limitations, and helps to control sensing limitations in applications.	[36]
2	Mesopores: Better mass transport	Mesopores (2–50 nm) help reduce diffusion resistance.		
3	Macropores: Improved bulk diffusion	Macropores (>50 nm) help for bulk diffusion and give accessibility for internal active sites.		
4	Hierarchical pores: Combines all advantages	It provides multiple sizes (2 nm to 50 nm), helping maintain a single framework.		
5	Enhances catalytic activity	Presence of polar groups and functionalized organic linkers.	Controls adsorption selectivity, which helps to separates gases.	[37]
6	Influences gas interactions (CO_2_, H_2_, VOCs, etc.)	open metal sites, acid/base groups, modified linkers		
7	Controls redox behavior	Nature of metal ions and bonding strength to organic linker	Determines hydrolytic and thermal stability, ensuring structural integrity under different environmental conditions.	[38]
8	Impacts long-term structural durability			
9	Critical for energy storage, sensing, electrocatalysis	Typically low in pristine MOFs; enhanced via design strategies	Can be improved using conjugated linkers, conductive guests/dopants, MOF–carbon hybrids, or by converting MOFs to derivatives	[39,40,41]
10	Excess defects reduce structural integrity and stability	Missing linkers, missing metal nodes, vacancies	Moderate defects create new active sites and boosts catalysis/adsorption	[40,41,42,43,44]

**Table 2:** Frequently used metal–organic freworks along with their stability, surface area ranges, commonly used functional linkers, and environmental applications.

MOFs Type	pH Stability and Water Stability	Surface Area	Common Functional Linkers	Major Environmental Applications	Reference
Zr-based	Ph-Stable in acidic, mildly basic conditions, but in water high hydrolytic stability	~800–1400 m^2^/g	BDC (terephthalate), amino-BDC, sulfonated linkers	Adsorption of heavy metals & dyes, CO_2_ capture, membrane separation, water treatment	[45]
Fe-based	Ph-Stable in mild acids, less stable in strong bases, but moderate water stability.	~1500–3500 m^2^/g	BTC (1,3,5-tricarboxylate), BDC, amino-linkers	Fenton-like catalysis for dyes/pharma, gas adsorption, pollutant degradation	[46]
Cu-based	Ph-Sensitive to high humidity & basic pH but water stability susceptible to moisture	~1500–3500 m^2^/g	BTC, Pyridine-based linkers.	VOC adsorption, gas separation, catalytic oxidation, low-humidity pollutant removal	[47]
Ni-based	Ph-Stable in neutral to mildly alkaline medium and water stability Moderate water stability.	~900–1300 m^2^/g	DOBDC (2,5-dihydroxyterephthalic acid), BDC derivatives	CO_2_ adsorption, electrocatalysis (HER/OER), heavy-metal adsorption	[48]

**Table 3:** Comprehensive Evaluation of MOFs.

Application	Properties	Features	Examples	Ref.
Adsorption & Separation	Tunable pore sizes & high surface area	Pores allow high adsorption capacity	MOFs with amino/hydroxyl functional groups coordinate heavy metal ions (As, Pb, Cd, Cr)	[55]
	Functional group modification	open-metal sites for selective binding	Dyes and pharmaceuticals removed via MOF composites or MOF-modified membranes	[56]
	Gas capture & separation	open metal sites, pore size tuning for CO_2_, NO_x_, SO_x_	CO_2_/N_2_ selectivity, VOCs capture from emissions	[57]
	Mixed-Matrix Membranes (MMMs) &MOF-membrane hybrids	MOF + polymer improves selectivity, reduces fouling, enhances transport	Membrane applications for desalination, gas purification	[58]
Catalytic Degradation of Pollutants	Photocatalysis	MOFs absorb UV/visible light, trigger electron-hole separation.	Ti-based (e.g., MIL-125), Zr-based (e.g., UiO-66), Zn-based (e.g., MOF-5) MOFs show semiconductor behavior	[59]
	Fenton-like & AOPs	Fe-MOFs activate H_2_O_2_ to generate ROS (hydroxyl, superoxide)	Degradation of persistent organics in water	[60]
	Composite/Heterojunction formation	MOF + semiconductor/nanoparticle hybrid improves charge separation	Improves efficiency and makes catalytic action more practical.	[61]
Sensing & Monitoring	Luminescent MOFs (LMOFs)	Fluorescent linkers or guest dyes, signal changes on analyte binding	Detect heavy metals (Fe^3+^, Cu^2+^, Hg^2+^, Pb^2+^, Cr(VI)), trace levels (ppb)	[62]
	Conductive MOF/MOF-polymer sensors	Chemi resistive, electrochemical responses improved by composite with polymers or conductive guests	Heavy metal ion sensing, gas sensing in environment	[63]
	Gas sensors	MOF films integrated into devices for CO_2_, NH_3_, H_2_ sensing; changes in conductivity/permittivity on gas adsorption	Metal–organic framework-based gas sensors	[57]
Antimicrobial & Disinfection Applications	Metal ion release & membrane disruption	MOFs loaded or constructed with Ag, Cu, Zn ions, which disrupt microbial membranes	Good for water/air disinfection filters/coatings	[64]
	ROS generation & entrapping microbes	MOFs generate ROS, or physically entrap pathogens in porous structure improving contact	Combined action enhances antimicrobial efficacy	[65]
	Carrier for sustained release	MOFs act as reservoirs for antimicrobial agents (ions, drugs) for controlled release	Useful for wound dressings, filters	[66]

**Table 4:** Details of key pollutants, commonly used MOFs for absorptions, adsorption capacity, and selectivity of MOFs, typical aqueous conditions.

Pollutant	Common MOFs Used for Absorptions	Adsorption Capacity	Selectivity Features	Reference
Arsenic	UiO-66-NH_2_, MIL-53(Fe), ZIF-8 composite, β-MnO_2_	50–150 mg/g (As(V)) 30–90 mg/g (As(III))	Strong affinity via –NH_2_/–OH groups; Zr-O clusters highly selective for oxyanions	[73]
Chromium	UiO-66-NH_2_, MIL-101(Fe), ZIF-8	100–300 mg/g	Electrostatic attraction at acidic pH (4 to 6); amino groups enhance reduction to Cr(III)	[74]
Heavy Metals	MOF-74(Ni), MIL-100(Fe), ZIF-67	80–250 mg/g	π-interactions & metal–ligand coordination drive selectivity	[75]
Dyes (anionic/cationic)	MIL-101, UiO-66 derivatives, ZIF-8, COF-MOF hybrids	200–1000 mg/g depending on dye	Size-selective adsorption, π–π stacking, electrostatic attraction	[76]
Emerging Organic Pollutants	UiO-66-NH_2_, NH_2_-MIL-53, MIL-100(Fe)	150–450 mg/g	Strong binding through amino and carboxyl groups	[77]

**Table 5:** Overview of energy device application and MOFs’ properties.

Application	MOFs Properties	Examples	Reference
Batteries	Carbons serve as high-surface-area anodes/cathodes for Li-ion, Na-ion, and Zn-ion batteries.	Higher capacity, better conductivity, and improved cycling stability.	[100]
Supercapacitors	Provide porous structures and redox-active centers enabling fast ion diffusion and charge storage.	High power density and rapid charge–discharge rates.	[101]
Electrocatalysis (HER/OER/ORR)	Act as catalysts or precursors for metal/metal-oxide catalysts used in hydrogen/oxygen evolution & reduction.	Tunable coordination sites improve catalytic efficiency.	[102]
Fuel Cells	Catalysts enhance hydrogen oxidation and oxygen reduction reactions in PEM fuel cells.	High catalytic activity with reduced noble-metal loading.	[103]
Redox Flow Batteries	Function as porous electrodes or redox-active frameworks improving charge transfer.	Increased electrolyte accessibility and high-rate capability.	[104]
Photoelectrochemical (PEC) Cells	Enhance light harvesting, charge separation, and catalytic water splitting efficiency.	Improved solar-to-hydrogen conversion efficiency.	[105]

## Data Availability

No new data were created or analyzed in this study. Data sharing is not applicable to this article.
